# Lipid droplet formation induced by icaritin derivative IC2 promotes a combination strategy for cancer therapy

**DOI:** 10.1186/s13020-024-01050-5

**Published:** 2024-12-26

**Authors:** Guosheng Wu, Liang Ying, Qian Zhang, He Xiong, Jie Wang, Sitao Chen, Chen Yang, Yiyuan Jin, Zengwei Lai, Ninghan Feng, Yunjun Ge

**Affiliations:** 1 MOE Medical Basic Research Innovation Center for Gut Microbiota and Chronic Diseases, Wuxi School of Medicine, Jiangnan University, Wuxi, China; 2 Department of Urology, Jiangnan University Medical Center, Wuxi, China; 3 School of Life Sciences and Health Engineering, Jiangnan University, Wuxi, China; 4 Suzhou Hospital of Anhui Medical University (Suzhou Municipal Hospital of Anhui Province), Suzhou, Anhui China; 5 Taizhou Center for Disease Control and Prevention, Taizhou, China

**Keywords:** Icaritin derivative, Anti-tumor, Lipid droplet, Mitochondrial function, DGAT1

## Abstract

**Background:**

Lipid metabolism is crucial in cancer progression. Lipid droplets (LDs) generated in cancer cells can act as protective mechanisms through alleviating lipotoxicity under stress conditions. We previously developed IC2 from the Chinese medicine icaritin as an inhibitor of stearoyl-CoA desaturase 1 (SCD1). IC2 has been shown to disrupt lipid metabolism and inhibits cancer cell proliferation. However, the impact of IC2 on intracellular LDs and the potential of targeting LD formation for combination cancer therapy remain unexplored.

**Methods:**

LD formation in cancer cells was analyzed with oil red O or BODIPY staining by microscopy. LD quantification was normalized to the cell number. IC2-induced cellular responses were revealed by transcriptional analysis, real-time PCR, and immunoblotting. Mitochondrial functions were assessed by measuring ATP production and oxygen consumption. The lipid source for LD formation was studied using lipid transporter inhibitors or lipid deprivation. The effect of inhibiting LD formation on IC2's anti-tumor effects was evaluated using MTT assays and apoptosis assays, which was subsequently validated in an in vivo xenografted tumor model.

**Results:**

IC2 exerted anti-tumor effects, resulting in LD formation in various cancer cells. LD formation stimulated by IC2 was independent of extracellular lipid sources and did not result from increased de novo fatty acid (FA) synthesis within the cancer cells. Transcriptional analysis indicated that IC2 disturbed mitochondrial functions, which was confirmed by impaired mitochondrial membrane potential (MMP) and reduced capacity for ATP production and oxygen consumption. Moreover, IC2 treatment led to a greater accumulation of lipids in LDs outside the mitochondria compared with the control group. IC2 inhibited the proliferation of PC3 cells and promoted the apoptosis of the cancer cells. These effects were further enhanced after inhibiting the diacylglycerol acyltransferase 1 (DGAT1), a key intracellular enzyme involved in LD formation. In PC3-xenografted mice, the DGAT1 inhibitor augmented the IC2-induced reduction in tumor growth by modulating LD formation.

**Conclusion:**

LD formation is a feedback response to IC2’s anti-tumor effects, which compromises the anti-tumor actions. IC2’s anti-tumor efficacy can be enhanced by combining it with inhibitors targeting LD formation. This strategy may be extended to other anti-tumor agents that regulate lipid metabolism.

## Introduction

Lipid metabolism plays a pivotal role in the initiation and progression of cancer, serving as a critical factor in sustaining the neoplastic transformation and supporting the cancer's aggressive behavior [1]. The reprogramming of lipid metabolism in cancer cells is characterized by an increase in de novo lipogenesis, and alterations in lipid uptake [2]. This metabolic shift not only fuels the biosynthesis of cellular membranes required for rapid proliferation but also provides a source of energy and bioactive lipids that contribute to tumor growth and survival [3]. Acetyl-CoA carboxylase 1 (ACC1) and SCD1 are enzymes responsible for catalyzing de novo FA synthesis, while DGAT1 plays a key role in catalyzing the formation of triacylglycerols (TGs) during the lipogenesis process [4].

Within lipid metabolism landscape, LDs are intracellular organelles for lipid storage, which are commonly observed with aggressive cancers [5]. In cancer cells, LDs provide a reservoir of lipids that are utilized for the synthesis of new membrane components, facilitating the rapid growth and proliferation of cancer cells. Additionally, LDs have other multiple functions in various cellular processes, including energy homeostasis, intracellular signaling, and stress responses [6]. Specifically, LDs can protect cancer cells against lipotoxicity, which may be caused by an imbalance between saturated and unsaturated FAs [7–9]. Therefore, LD formation is an active adaptive mechanism that cancer cells exploit to promote their survival and progression [5].

The generation of intracellular LDs has much relationships with mitochondrial functions. Mitochondria of the LD-bound type, which have an excessive capacity for lipid biogenesis, can directly promote LD formation in adipocytes [10]. Mitochondrial dysfunction can disrupt lipid transportation through the intracellular compartments and lead to LD formation in enterocytes [11]. Mitochondrial functions depend on the mitochondrial encoded proteins, such as ND5, CO2, and ATP8, and are also influenced by nucleus-coded proteins, such as ID1, PLK1, and CDK1 [12–15]. The transcription of mitochondrial encoded proteins is regulated by the proliferator-activated receptor-γ co-activator 1α (PGC-1α) and mitochondrial transcription factor A (TFAM), which have been recognized as two master regulators of mitochondrial functions [16]. Dysregulation of these key factors related to mitochondrial functions has been shown to regulate the formation of intracellular LDs [17, 18].

Icaritin, a naturally occurring prenylated flavonoid derived from the Chinese herb Epimedium, has demonstrated promising anti-tumor effects against various cancers, such as hepatocellular carcinoma [19]. Icaritin inhibited the proliferation of tumor cells by inducing cell apoptosis, which was accompanied by mitochondrial autophagy [20]. And inhibiting the process of mitochondrial autophagy could enhance the anti-tumor effects of icaritin. The combination of natural products with other agents that can overcome drug resistance mechanisms has been recognized as a powerful strategy for cancer treatment [21].

Our research group has developed an icaritin derivative, IC2, with potential anti-tumor effects on breast cancer by inhibiting SCD1 [22]. IC2 could induce the apoptosis of breast cancer cells, accompanied by activating the activation of adenosine monophosphate (AMP)-activated protein kinase (AMPK) signaling and stimulating cytoprotective autophagy, and inhibiting AMPK-mediated autophagy could enhance the anti-tumor effects of IC2 [23]. However, the effects of IC2 on mitochondrial functions and LD formation in cancer cells remain to be explored.

In this study, we found that the anti-tumor agent, IC2, stimulated LD formation in various tumor cells. IC2-stimulated LD formation was mostly caused by the disruptions of mitochondrial functions and relied on the functions of ACC and DGAT1 in cancer cells. The anti-tumor effects of IC2 could be enhanced through combining it with inhibitors that target LD formation pathways.

## Materials and methods

### Cell culture and materials

PC3, LNCaP, A549, and MDA-MB-231 cell lines were obtained from the National Collection of Authenticated Cell Cultures (Shanghai, China). PC3 and LNCaP cells were cultured in RPMI 1640 medium supplemented with 10% fetal bovine serum (FBS) at 37 °C in 5% CO_2_. MDA-MB-231 and A549 cells were cultured in DMEM medium supplemented with 10% FBS at 37 °C in 5% CO_2_.

Icaritin was purchased from Nanjing Spring & Autumn Biotech (Cat: E-0846; Nanjing, China). FBS (Cat: F7524) was sourced from Sigma (St. Louis, MO, USA). The MTT assay kit (Cat: MB4698) was obtained from Meilunblo (Dalian, China). The BCA Protein Assay Kit (Cat: 23227) and BODIPY 558/568 C12 (Cat: D3835) were purchased from Thermo Fisher (Waltham, MA, USA). The Oil Red O Staining Kit (Cat: C0157S), Mito-Tracker Red CMXRos (Cat: C1035), Mito-Tracker Green (Cat: C1048), and ATP Assay Kit (Cat: S0026) were sourced from Beyotime (Shanghai, China). BODIPY 493/503 (Cat: HY-W090090) was purchased from MedChem Express (New Jersey, USA). The Triglyceride assay kit (Cat: A110-1-1) and Total cholesterol assay kit (Cat: A111-1-1) were acquired from Nanjing Jiancheng Bioengineering Institute (Nanjing, China). The Oxygen Consumption Rate Assay Kit was obtained from Cayman (Cat: 600800; Ann Arbor, MI, USA). The FITC Annexin V Apoptosis Detection Kit was sourced from BD Biosciences (Cat: 556547; Becton Dickinson and Company, San Jose, CA, USA). The Chemiluminescent Substrate Kit (Cat: 36222ES76) was purchased from Yeasen Biotech (Shanghai, China).

### IC2 synthesis

Icaritin was dissolved to its maximum solubility in 150 mL of acetone with 1-bromo-3-methyl-2-butene. The reaction was initiated by adding 300 mg of anhydrous potassium carbonate to the mixture. The solution was stirred for 4 h at 56 °C to facilitate the reaction. Subsequently, the reaction mixture was concentrated under reduced pressure, and the pH was adjusted to 4–5 using 1 N HCl. The resulting products were extracted three times with 100 mL of dichloromethane. The organic extracts were then dried using anhydrous sodium sulfate, filtered, and concentrated under reduced pressure. IC2 was further purified from the resulting residue through silica gel column chromatography using a mixture of petroleum ether and ethyl acetate in a ratio of 10:1.

### Cell viability assay

The cell viability was tested by using the MTT assay according to the manufacturer’s protocol. In brief, 8000 cells per well were seeded in a 96-well microplate overnight and incubated with compounds at different concentrations for 24 h. Per well was added with 20 μL MTT reagent and incubated for 4 h at 37 °C. Afterward, the supernatant was discarded, and 150 μL dimethyl sulfoxide was added to cells for 10 min. The light absorbance value was measured at 490 nm using a BioTek Synergy H4 microplate reader (Vermont, USA). The results of the treated groups were normalized to the control group:

$${\text{Cell}}\;{\text{viability}}\;{\text{of}}\;{\text{each}}\;{\text{group}} = \left( {{\text{treated}}\;{\text{group}}\;{\text{absorbance}} - {\text{blank}}\;{\text{group}}\;{\text{absorbance}}} \right)/\left( {{\text{control}}\;{\text{group}}\;{\text{absorbance}} - {\text{blank}}\;{\text{group}}\;{\text{absorbance}}} \right).$$

### Oil red O staining of LDs

Cells were inoculated in 6-well plates, with each group assigned two wells (one for oil red O staining and the other for protein concentration detection for quantification). After treatment with IC2, the cells were washed with PBS, fixed with 4% paraformaldehyde for 15 min, and stained with oil red O staining solution for 30 min. Images were captured using the Axio Vert.A1 microscope (Carl Zeiss; Oberkochen, Germany). To quantify the content of intracellular LDs, the stained cells were treated with 100% isopropyl alcohol to extract the oil red O for 10 min, and the LD amount was then measured by absorbance at 510 nm. The total protein amount in the corresponding wells was measured using the BCA kit, with absorbance read at 562 nm. The relative LD level was calculated using the following formula:

$${\text{Relative}}\;{\text{LD}}\;{\text{level}} = {\text{oil}}\;{\text{red}}\;{\text{O}}\;{\text{amount}}/{\text{protein}}\;{\text{amount}}.$$

### MMP analysis

The cells were cultured in glass-bottom dishes and treated with different concentrations of IC2 for 24 h. MMP was analyzed by staining the cells with the MitoTracker Red CMXRos for 30 min according to the manufacturer’s instructions. Cell nuclei were stained with 1 μg/mL Hoechst for 30 min followed by PBS washes. Cell images were captured with the Inverted Zeiss LSM880 laser scanning confocal microscope (Oberkochen, Germany).

### Measurement of ATP production

Cells were stimulated with or without 20 μM IC2 for 24 h. ATP production was analyzed using the ATP Assay Kit, in accordance with the manufacturer’s instructions. Briefly, the cells were collected on ice and immediately lysed with the assay kit. After being centrifuged at 12,000×*g* for 5 min at 4 °C, the supernatant was collected for the determination of ATP with the Biotek Synergy H4 plate reader (Vermont, USA). ATP standard curve was established using a gradient concentration of ATP from 0.1 to 10 µM. The ATP concentrations in the samples were normalized to the amount of proteins measured by a BCA kit.

### Analysis of oxygen consumption

Intracellular oxygen consumption was detected using the Oxygen Consumption Rate Assay Kit. For each well, 1 × 10^6^ cells in 200 μL of culture media were inoculated into a 96-well clear-bottom plate. After treatment with IC2, cell culture medium was replaced with medium containing the phosphorescent oxygen probe. The samples were then covered with mineral oil to seal off the air supply, and kinetic measurements were initiated immediately at an excitation wavelength of 380 nm and an emission wavelength of 650 nm, recorded every 2 min for 2 h at 37 °C using the Biotek Synergy H4 plate reader.

### Distribution of intracellular FAs

To analyze the distribution of intracellular FAs, cells were labelled for 16 h with 1 mM BODIPY 558/568 C12 during IC2 treatment. Mitochondria was labeled with 200 nM MitoTracker Green for 30 min prior to imaging. For LD labelling, BODIPY 493/503 was added to cells at 5 μM prior to imaging. The images were captured with the Zeiss LSM880 confocal microscope. Colocalization of BODIPY 558/568 C12 and LD/mitochondria was assessed via Colocalization Finder using the ImageJ software (Rawak Software Inc, Germany).

### TG and cholesterol measurement

Triacylglycerols (TGs) and total cholesterol (T-CHO) contents in cancer cells or tumor samples were measured using the TG assay kit and Total cholesterol assay kit, respectively. In brief, the cells or tumors were lysed with ultrasonic apparatus. The detection kit reagent was added to the samples, which were loaded in 96-well plates. The plates were kept at 37 °C for 10 min in the dark. Finally, the optical density was measured at 510 nm using the Biotek Synergy H4 plate reader. Protein concentration as a control was detected using the BCA Kit.

### Flow cytometry of cell apoptosis

To analyze cell apoptosis, the Annexin V-FITC Apoptosis Detection Kit was utilized. After incubation with IC2 in combination with T863 for 24 h, cancer cells were harvested using EDTA-free trypsin and then resuspended with binding buffer. The cells were stained with Annexin V-FITC and propidium iodide (PI) for 15 min at room temperature in the dark according to the manufacturer’s instructions. The stained cells were analyzed by the BD C6 Flow Cytometer. The cell populations of early and late apoptotic cells were collectively considered as apoptotic cells. The percentages of these two populations were combined, and the resulting values are presented.

### Quantitative real-time PCR

After IC2 treatment, total RNA was isolated using the RNAiso plus kit, and cDNA was prepared through reverse transcription. Expression of mRNA was quantified with the LightCycler 480 System (Roche; Basel, Switzerland) using the SYBR Green I Master, following the manufacturer’s instructions. The expression of target genes was analyzed by the 2-∆∆Ct method with β-actin as an internal control. The following PCR primers were used: β-actin, 5′-TGACGTGGACATCCGCAAAG and 5′-CTGGAAGGTGGACAGCGAGG; PGC-1α, 5′-GATGACAGCGAAGATGAA and 5′-GAAGAACAAGAAGGAGACA; TFAM, 5′-CTCCAAGTCAGATTATGT and 5′-TTCCTTTACAGTCTTCAG.

### Western blotting

The cancer cells in 12-well plates were treated with IC2 and/or other compounds, followed by lysis in RIPA buffer supplemented with protease inhibitors. For tumor samples, 20 mg of mouse tumors were homogenized and lysed in a similar manner to prepare samples for analysis. Protein concentration of the lysates was determined using BCA Protein Assay Kit. Protein samples were loaded for electrophoresis on SDS–PAGE and transferred to PVDF membranes. The membranes were blocked with 5% non-fat milk for 2 h at room temperature, and then incubated with primary antibodies overnight at 4 °C. The membranes were then incubated with horseradish peroxidase-conjugated secondary antibodies (1:5000; Cell Signaling Technology, 7074S) for 2 h at room temperature. The blots were detected using chemiluminescence detection buffer with the ChemiDoc MP Imaging System (Bio-Rad; Hercules, USA).

The usage and supplier information for the primary antibodies are as follows: PGC-1α (1:1000; Proteintech, 66,369–1-Ig), TFAM (1:1000; Proteintech, 23996-1-AP), β-actin (1:2500; Abclonal, AC006), SREBP1 (1:1000; Proteintech, 14088-1-AP), FASN (1:1000; Proteintech, 10624-2-AP), ACC (1:1000; Cell Signaling Technology, 3676), β-tublin (1:2500; Abclonal, AC008), PARP (1:1000; Cell Signaling Technology, 9532), caspase-3 (1:1000; Proteintech, 19677-1-AP) and cleaved caspase-3 (1:1000; Cell Signaling Technology, 9664).

### Xenograft mouse model

Animal experiments were conducted with the approval of the Experimental Animal Care and Use Committee at Jiangnan University, following the guidelines of the World Medical Association (WMA) Statement on animal use in biomedical research. The approval number is JN.NO2023103060560220[497]. Male BALB/c nude mice aged between 4 to 6 weeks were obtained from SPF Biotechnology Co., Ltd. (Beijing, China). PC3 cells (5 × 10^6^) were subcutaneously inoculated into the nude mice. When tumor volumes reached between 150 and 300 mm^3^ after about 2 weeks, the mice were randomly allocated into three groups. Treatments were administered once every 2 days via intratumoral injections, using either PBS, IC2 (20 μM), or a combination of IC2 (20 μM) and T863 (20 μM). Tumor progression was tracked by measuring tumor dimensions in length (L) and width (W), with tumor volumes (TV) calculated using the formula: TV = 0.5 × L × W^2^. Additionally, the body weights of all mice were recorded throughout the treatment period. Tumor samples were collected following mouse sacrifice at the end of the experiment. Mice tumors were cryo-sectioned and stained with oil red O at described above.

### Statistical analysis

The results obtained were based on at least three independent experiments, and the data were presented as means ± SEM. To compare the means between two groups, the Student’s *t*-test was employed. For multiple comparisons, one-way analysis of variance (ANOVA) was used to assess the differences in means among groups. All data were analyzed using GraphPad Prism 7.0 software (GraphPad Software, San Diego, CA, USA), unless otherwise specified. *P*-value less than 0.05 was considered statistically significant.

## Results

### IC2 exerted anti-tumor effects and promoted LD formation in cancer cells

**Fig. 1:**
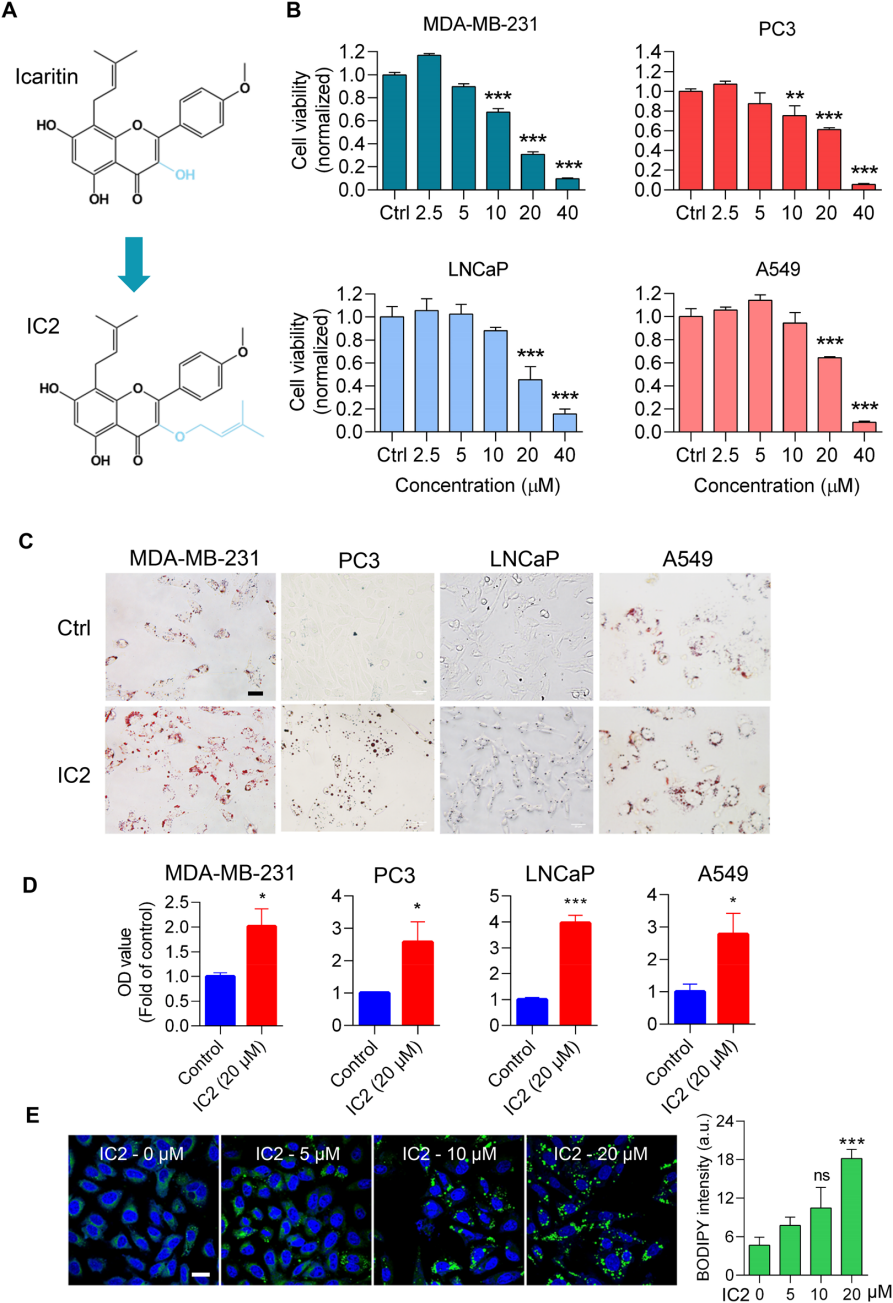
IC2 inhibited cancer cell proliferation with the generation of LD. **A** Chemical structure of icaritin and IC2. **B** Inhibition of the proliferation of various cancer cells. The cancer cells were treated with IC2 at different concentrations for 24 h. Cell viabilities were analyzed with MTT assays. **C** Microscopy of intracellular LDs with Oil red staining. The indicated cells were treated with 20 μM IC2. Representative images from three independent experiments were shown. **D** Quantification of LD staining. The quantification was determined by dividing the oil red staining by the BCA staining of total protein for each sample. **E** Confocal microscopy of LDs with BODIPY 493/503 staining. PC3 cells were treated with different concentrations of IC2 for 24 h. Representative images were shown, with statistical analysis on the right. The symbol “a.u.” signifies arbitrary units. Scale bar = 20 μm. *P < 0.05, **P < 0.01 and ***P < 0.001

As previously reported, IC2 is a semi-synthetic compound originating from the traditional Chinese medicine, icaritin (Fig. 1A). Besides its effects against breast cancer, the anti-tumor properties of IC2 were explored in other types of cancer, including prostate and lung cancers. In MTT assays, IC2 demonstrated the capability to inhibit the viability of prostate cancer (PCa) cell lines, PC3 and LNCaP, as well as the lung cancer cell line, A549, in addition to the breast cancer cell line, MDA-MB-231 (Fig. 1B). Overall, the inhibitory effects of IC2 exhibited a dose-dependent manner, with a concentration of 20 μM being effective against most cancer cell types.

Given that IC2 has been reported to inhibit SCD1 activity, we speculated that interference with the lipid synthesis process in cancer cells may cause an imbalance between saturated and unsaturated FAs, potentially leading to lipotoxicity. Consequently, we analyzed intracellular LDs, organelles frequently observed in cancer cells that function in lipid storage and alleviating lipotoxicity. The cancer cells were treated with IC2 and subsequently stained with oil red O. Microscopic analysis revealed that more LDs in cancer cells were observed, as compared to the untreated cells, suggesting IC2 promoted LD formation (Fig. 1C). Quantification of LD staining was performed by normalizing LD staining to the amount of total cell protein. The quantification results confirmed that IC2 promoted LD formation in all of the cancer cells tested (Fig. 1D). PC3 cells were further stained with BODIPY 493/503, a fluorescent dye commonly used for LD staining. Confocal microscopy analysis also demonstrated IC2 promoted LD formation in the cancer cells (Fig. 1E). Therefore, the induction of LDs in tumor cells appears to be a common event that accompanies with the anti-tumor effects of IC2.

### IC2-promoted LD formation was independent of extracellular lipids

**Fig. 2:**
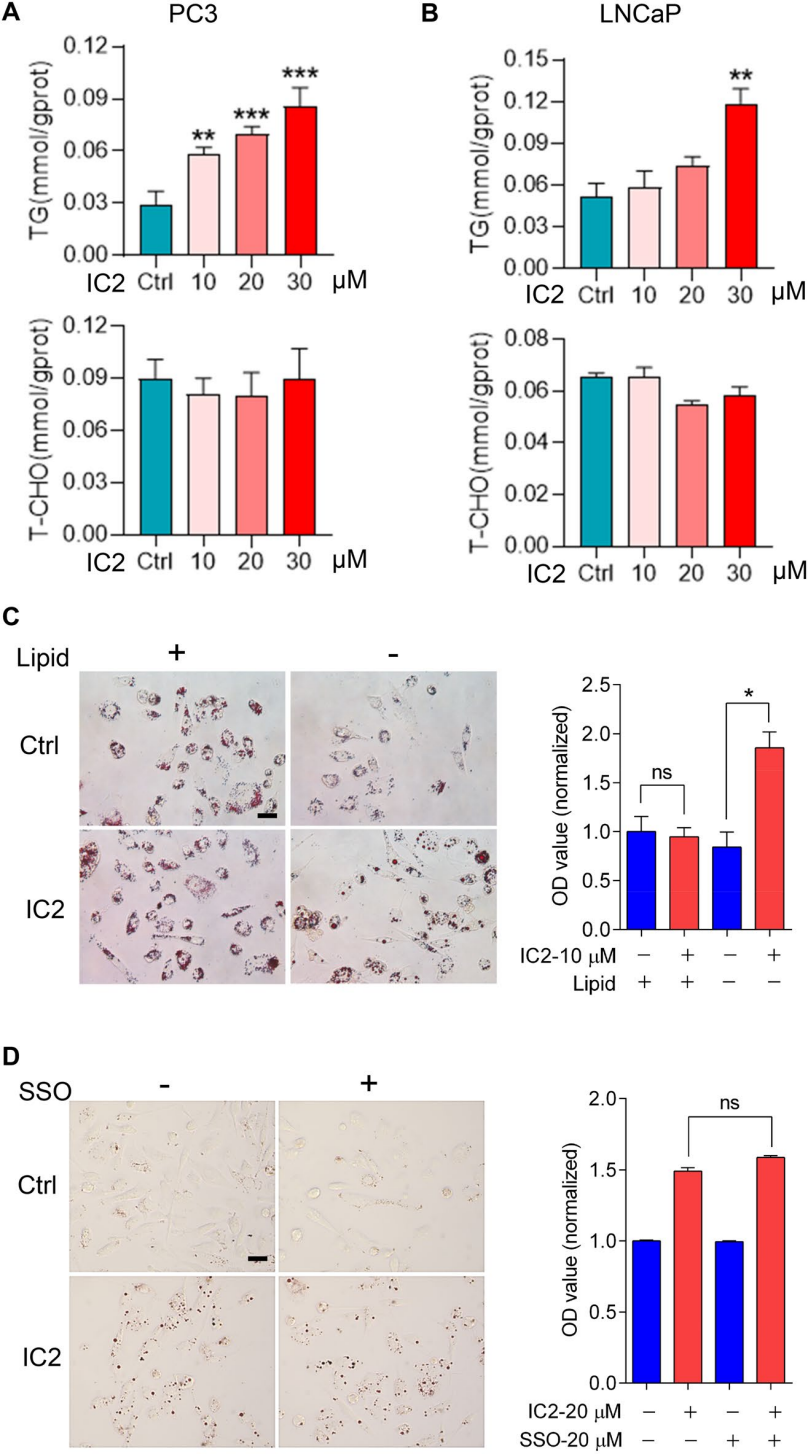
IC2-stimulated LD formation did not depend on extracellular lipids. **A** Analysis of lipid composition in LDs in PC3 cells. The cells were stimulated with the indicated concentrations of IC2 for 24 h. Intracellular LDs were collected and analyzed. **B** Analysis of the LDs in LNCaP cells. The cells were treated and analyzed as above. **C** IC2-stimulated LD generation after lipid depletion. Cells were cultured in lipid-free medium, stimulated with 10 μM IC2, stained with oil red O and observed under microscope. The quantification was based on oil red O staining relative to total protein staining. **D** Influences of CD36 inhibition on LD formation. Cells were treated with or without 20 μM SSO and LD formation stimulated by 20 μM IC2 was analyzed as above. Quantification of LDs with CD36 inhibition were conducted as above. Representative images from three independent experiments were shown. Scale bar = 20 μm. *P < 0.05, **P < 0.01 and ***P < 0.001

The mechanisms underlying IC2-stimulated LD formation were investigated to revealed its roles in the anti-tumor effects of IC2. We first analyzed the lipid composition of IC2-stimulated LDs in the cancer cells. As PCa is probably more relevant to lipid metabolism compared to other cancers [24], we chose the PCa cell line, PC3, as a typical cell model in subsequent studies. The levels of TGs and T-CHO were measured using corresponding ELISA kits. In PC3 cells, IC2 treatment increased the levels of TGs in a dose-dependent manner, while the levels of T-CHO were not significantly affected (Fig. 2A). IC2-stimulated intracellular changes with TG increase were also observed in LNCap cells, suggesting this result may be consistent in other cancer cells (Fig. 2B).

We then proceeded to investigate the sources of lipids for LD formation. Extracellular lipids were depleted by culturing the cancer cells in a lipid-free medium. Under this condition, IC2 was able to stimulate LD formation at a concentration of 10 μM, which was not effective in inducing LD formation when lipids were present with the normal medium (Fig. 2C). When the concentration of IC2 was increased to 20 μM or higher in the absence of extracellular lipids, cell viability was significantly compromised, resulting in the loss of cancer cells that could be stained for LD imaging or quantification (data not shown). Nonetheless, our results suggest that IC2-induced LD formation is not dependent on extracellular lipids.

The cell surface transporter, CD36, has been identified as a crucial FA importer for cancer cells [25]. Sulfosuccinimidyl oleate (SSO) was applied to block the FA translocase, CD36. Treatment with SSO did not significantly affect LD formation promoted by IC2 (Fig. 2D). Quantitative analysis of LD content in the cancer cells confirmed that the CD36 inhibitor did not impede the IC2-induced LD formation (Fig. 2D). Therefore, IC2-stimulated LD formation in cancer cells is not dependent on extracellular lipids.

### IC2 effects had some relationships with lipid metabolism-related enzymes

**Fig. 3:**
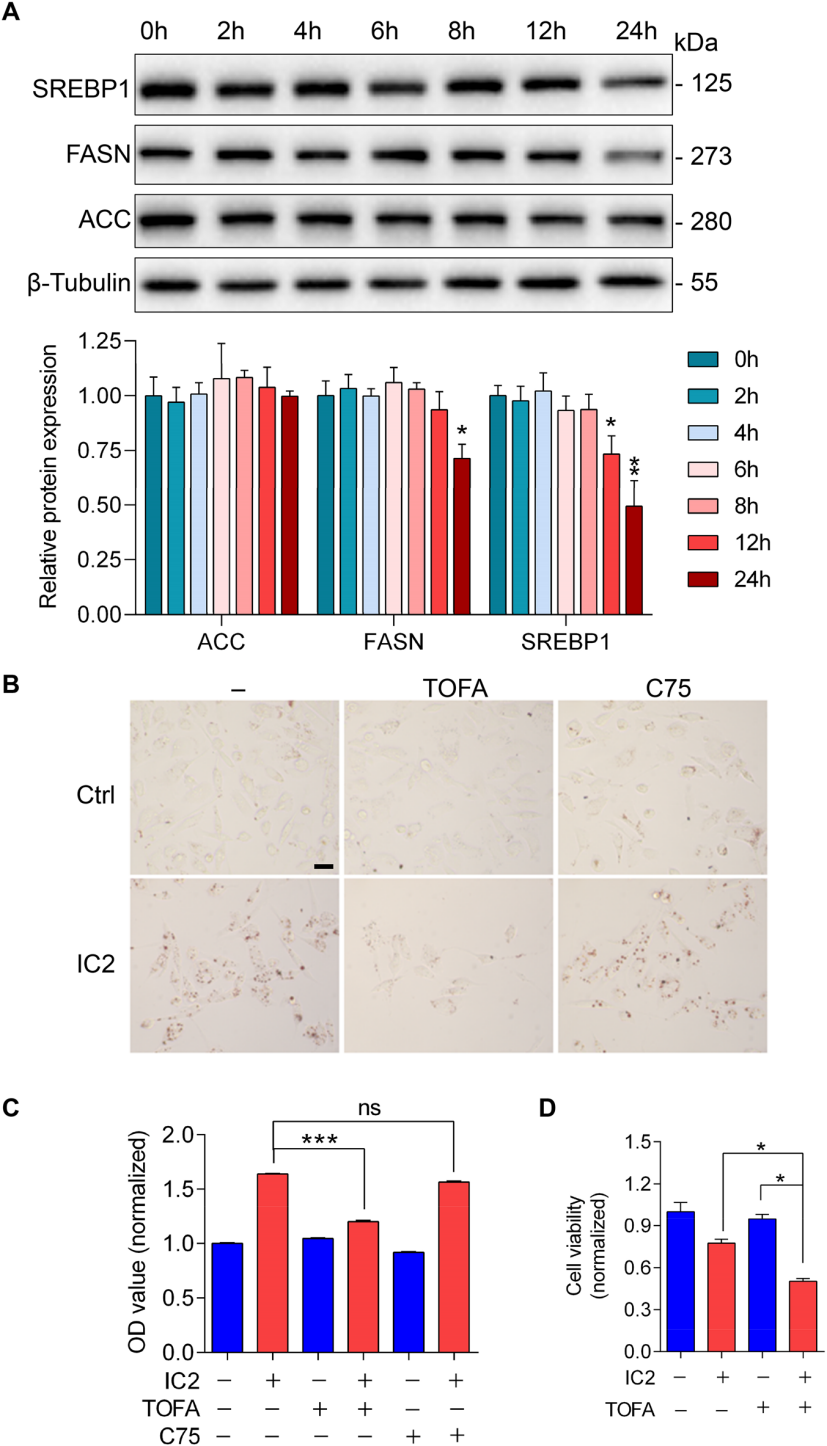
FA synthesis process was not accelerated for IC2-stimulated LD generation. **A** IC2 influences on FA synthesis enzymes. PC3 cells were treated with 20 μM IC2 for different time periods and the indicated proteins were analyzed by Western blotting. Relative protein levels were calculated based on blot intensities of the indicated proteins and the reference protein. **B** Effects of ACC or FASN inhibition on LD generation. PC3 cells were treated with 20 μM TOFA (ACC inhibitor) or 20 μM C75 (FASN inhibitor) plus 20 μM IC2. LDs in the cells were stained with oil red O and microscopy were conducted as above. **C** Quantification of LD formation in IC2-stimulated cells. The quantification was determined by dividing the oil red O staining by the BCA staining of total protein. Representative images from three independent experiments were shown. **D** Effect of ACC inhibition on cell proliferation. PC3 cells were treated with IC2 in the presence or absence of TOFA for 24 h. Scale bar = 20 μm. *P < 0.05, **P < 0.01 and ***P < 0.001

Since IC2-stimulated LD formation did not depend on extracellular lipids, we speculated that the source of lipids for LD formation in the cancer cells is derived from de novo FA synthesis. Consequently, the expression of enzymes related to FA synthesis in the process of LD formation were investigated. At different time points of IC2 treatment, the expression levels of FA synthesis-related enzymes, including acetyl-CoA carboxylase (ACC) and FA synthase (FASN), as well as the upstream regulatory element, the sterol regulatory element-binding protein 1 (SREBP1) did not increase (Fig. 3A). Instead, IC2 treatment reduced the expression of SREBP1 and FASN in PC3 cells, with ACC expression not significantly affected (Fig. 3A). These results are against the possibility that IC2 stimulates elevated expression of related enzymes to promote FA synthesis in the cancer cells.

The down-regulation of FASN during IC2-stimulated LD formation suggests that FA synthesis may not be necessary for the formation of LDs in the cancer cells. To confirm the roles of ACC and FASN in LD generation, we applied specific inhibitors of these enzymes plus IC2 treatment. When intracellular ACC was inhibited by 5-(tetradecyloxy)-2-furoic acid (TOFA), IC2-stimulated LD formation in PC3 cells was compromised, suggesting a possibility that FA synthesis is involved in LD formation (Fig. 3B and C). Interestingly, ACC inhibition enhanced the inhibitive effects of IC2 on cell proliferation (Fig. 3D). However, inhibition of FASN, the enzyme responsible for catalyzing the synthesis of the long-chain saturated FAs, did not reduce IC2-stimulated LD formation, excluding the possibility of the involvement of FA synthesis (Fig. 3B and C). The paradoxical phenomenon can be explained by the fact that TOFA acts as a pan-inhibitor of both ACC1 and ACC2 [26]. While ACC1 is responsible for FA synthesis, ACC2 plays a role in inhibiting FA oxidation in mitochondria [27, 28]. Therefore, the inhibitory effects of TOFA on IC2-stimulated LD formation may be attributed to ACC2 inhibition, which results in increased utilization of intracellular lipids by the mitochondria in cancer cells.

### Transcriptional analysis indicated IC2 stimulated mitochondrial disturbance

**Fig. 4:**
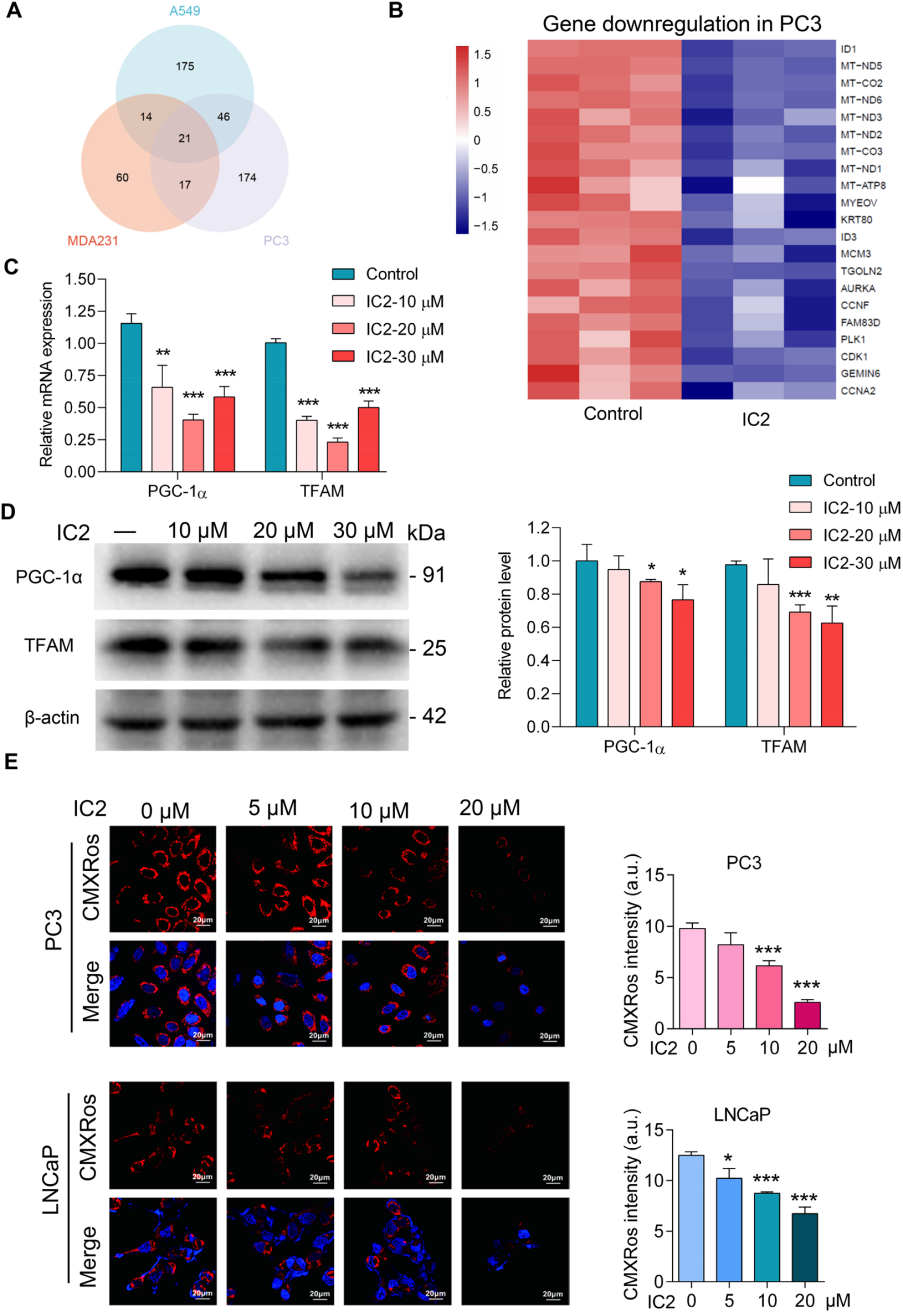
IC2 stimulated mitochondria disruption in various cancer cells. **A** Transcriptional analysis of changes in gene expression. The indicated cancer cells were treated with 20 μM IC2 for the analyses. **B** Down-regulated genes associated with IC2 treatment. Gene expression levels were represented by color intensities, and the left panel provided an explanation for the increase or decrease. **C** RT-qPCR analysis of the expression of mitochondria regulators. The mRNA samples were prepared from PC3 cells with or without IC2 treatment. **D** Western blotting analysis of the expression of mitochondria regulators. The cells were treated as above and protein samples were prepared. Protein expression levels were quantified based on the blot intensities from three independent experiments. **E** IC2 influences on MMP. The membrane potential is indicated by the red fluorescence, with statistical analysis on the right. CMXRos and Hoechst staining were merged. Representative images from three independent experiments were shown. *P < 0.05, **P < 0.01 and ***P < 0.001

To further explain LD generation, transcriptional analysis was conducted with IC2-treated cancer cells. IC2 treatment resulted in the down-regulation of numerous genes, with 21 genes being common to various types of cancer cells (Fig. 4A). These genes included mitochondrial genes, such as MT-ND1-3, MT-CO2-3, MT-ATP8, as well as some nuclear genes, such as ID1, PLK1, and CDK1 (Fig. 4B). These genes edit proteins that have much relationship with mitochondrial functions. Therefore, the results of transcriptional analysis indicate that mitochondrial disturbance might contribute to IC2-stimulated LD formation in the cancer cells. This speculation is also consistent with the results related to TOFA inhibition as mentioned above, which suggest that mitochondrial functions regulate LD formation.

As revealed in our previous study [23], IC2 stimulation resulted in AMPK activation. AMPK signaling affects the expression of two key regulators in mitochondrial functions, PGC-1α and TFAM. Therefore, the expression of these two regulators was analyzed with IC2 treatment. Real-time PCR analyses indicated that IC2 treatment led to the down-regulation of PGC-1α and TFAM in PC3 cells (Fig. 4C). The down-regulation was confirmed by Western blotting analyses of the expression of PGC-1α and TFAM at the protein level (Fig. 4D). These results indicated that IC2-induced down-regulation of PGC-1α and TFAM possibly explained the down-regulation of mitochondria-related genes as shown in Fig. 4B.

To examine whether the disturbance affected mitochondrial functions, the cancer cells were stained with Chloromethyl-X-rosamine (CMXRos), a red fluorescent dye that accumulates within mitochondria in the presence of a negative MMP. Microscopic analyses revealed that IC2 stimulation resulted in the loss of MMP in two of the PCa cell lines, indicating IC2 stimulated a disruption of mitochondrial functions (Fig. 4E).

### IC2 induced mitochondrial dysfunction and the storage of lipids in LDs

**Fig. 5:**
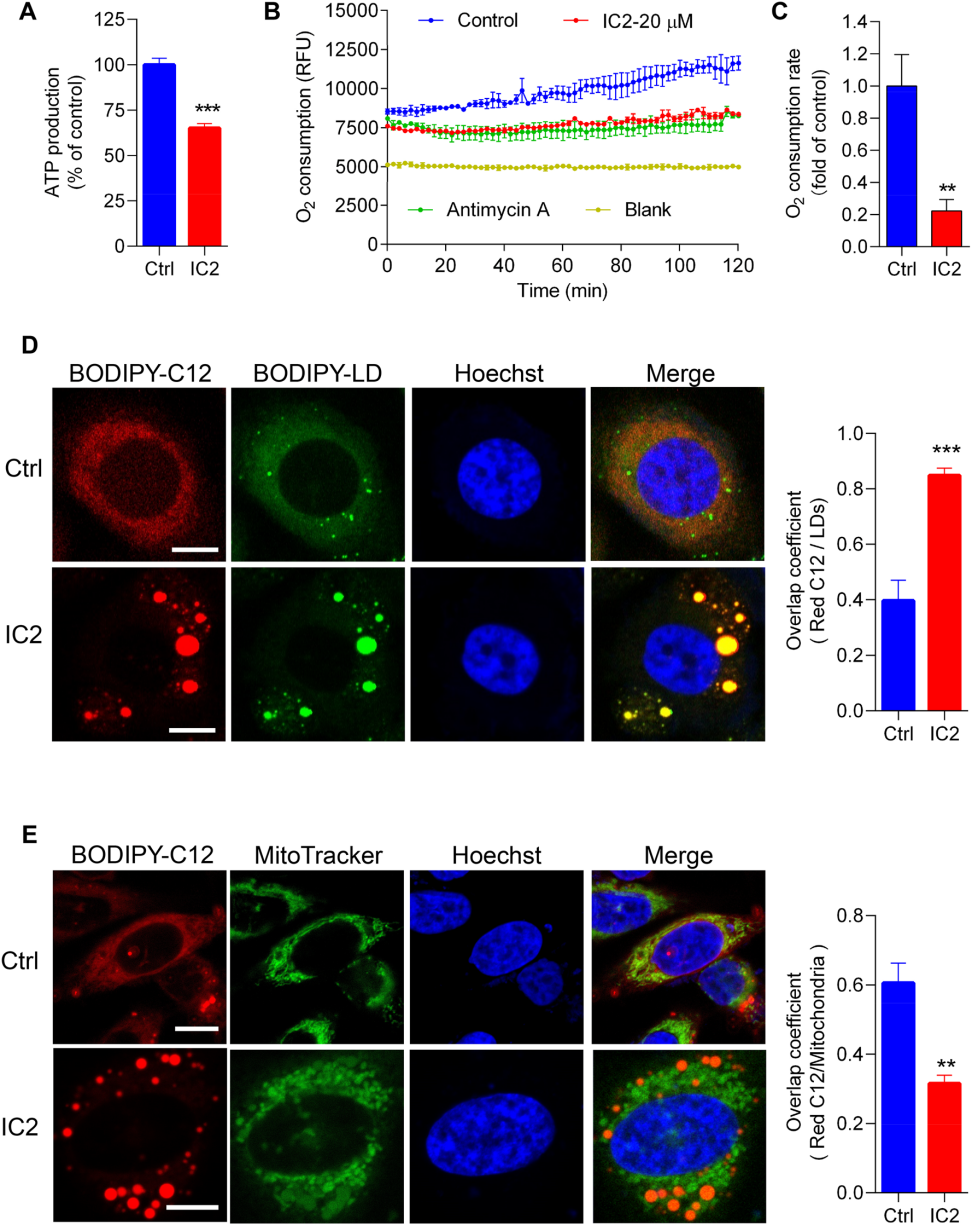
Mitochondria functions and lipid distribution in IC2-stimulated cancer cells. **A** ATP production in PC3 cells. The cells were treated with or without 20 μM IC2 and ATP concentrations were measured. **B** Realtime measurement of intracellular oxygen concentrations. Intracellular oxygen was analyzed with the Phosphorescent Oxygen Probe. Oxygen amount was inversely proportional to the signal. **C** Oxygen consumption rate in cancer cells. The rate was calculated as the changes in signals over time. **D** FA distribution in LD after IC2 treatment. Intracellular FAs were stained with BODIPY 558/568 C12 and shown in red. LDs were stained with BODIPY 493/503 and shown in green. Cell nuclei were stained with Hoechst and shown in blue. **E** FA distribution in mitochondria with IC2 treatment. The mitochondria were stained with the MitoTracker Green. Cell nuclei and LDs were stained as above. Representative images from three independent experiments were shown. Scale bar = 10 μm. **P < 0.01 and ***P < 0.001

To further examine IC2 influences on mitochondrial functions, ATP production and oxygen consumption in the mitochondria of cancer cells were analyzed. In comparison to untreated cancer cells, ATP production in IC2-treated cells was significantly decreased, suggesting inhibition of oxidative phosphorylation (Fig. 5A). Real-time measurement of oxygen levels inside cancer cells showed detectable oxygen consumption in untreated cells. IC2 treatment markedly reduced oxygen consumption in cancer cells compared to the untreated group (control), resembling the effect of the positive control, antimycin, a known inhibitor of oxygen consumption (Fig. 5B). Quantitative analysis of the oxygen consumption rate confirmed that IC2 impaired the oxygen consumption capacity of cancer cells (Fig. 5C). These results indicated that IC2 stimulation led to mitochondrial dysfunction in cancer cells.

The dysfunction of mitochondria implies a decreased utilization of FAs in IC2-treated cancer cells. To confirm this, FA distribution in the cancer cells were analyzed. PC3 cells were stained using BODIPY558/568-C12 (Red-C12), a fluorescent probe designed for staining all intracellular FAs [29]. Confocal microscopy revealed that the red dye (Red-C12) colocalized well with the LD staining dye, BODIPY 493/503 (BODIPY-LD), regardless of IC2 treatment (Fig. 5D). This indicated that most intracellular lipids were distributed in the LDs after IC2 treatment. Subsequently, cancer cells were stained with the mitochondria dye, MitoTracker Green. In untreated cells, most lipids were found to co-localize with the mitochondria, as observed in microscopy images of cells stained with Red-C12 and MitoTracker Green. Following IC2 treatment, a significant portion of lipids was located outside the mitochondria, potentially stored in LDs (Fig. 5E). Quantification of the microscopy images showed a decrease in the overlap coefficient, demonstrating that a significant amount of lipids was unable to be utilized in IC2-treated cancer cells (Fig. 5E). These observations reveal that IC2-stimulated LD formation is attributed to mitochondrial dysfunction, which reduces the utilization of intracellular lipids in cancer cells.

### Inhibiting LD formation enhanced the anti-tumor effects of IC2

**Fig. 6:**
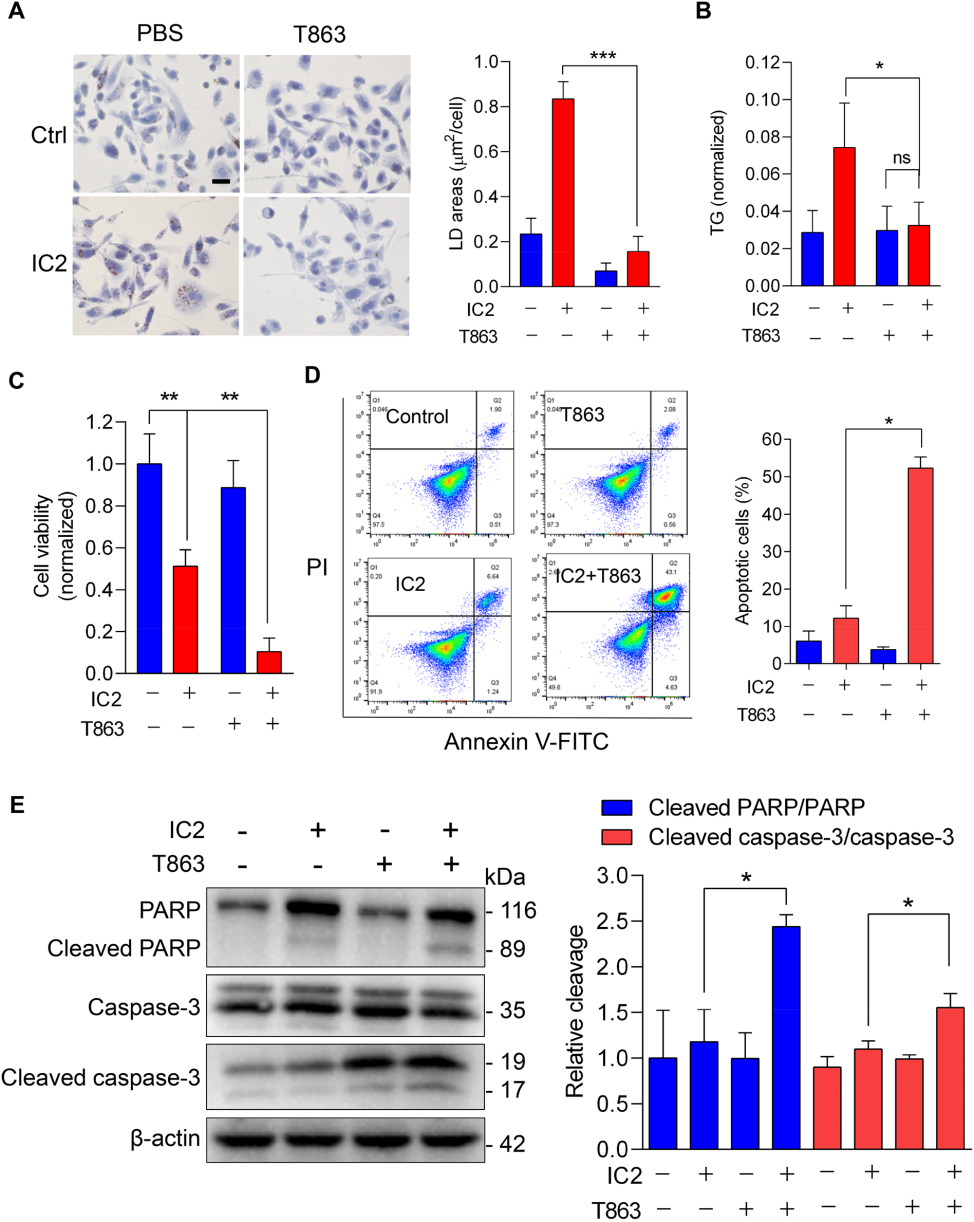
Combination of the DGAT1 inhibitor with IC2 to promote anti-tumor effects. **A** Reduced LD formation with DGAT1 inhibition. PC3 cells were stimulated with 20 μM IC2 in the presence or absence of 20 μM T863. LDs in the treated cells were stained by oil red O. Quantification of LD staining were shown in the right panel. Scale bar = 20 μm. **B** TG contents regulated by the DGAT1 inhibitor. Cells were treated as above. TG contents normalized to total protein amount were calculated. **C** Enhanced inhibition of cell viability with the DGAT1 inhibitor. Cell viability was analyzed with MTT assays. **D** Flow cytometry of cell apoptosis induced by IC2 combined with the DGAT1 inhibitor. The cells were treated as above and further stained with PI and Annexin V-FITC. Quantification of the apoptosis cells were shown in the right. **E** Immunoblotting analysis of cell apoptosis. PARP and caspase-3, as well as their cleaved forms, were analyzed after the cells were treated with IC2 plus T863. Relative cleavage of PRAR or caspase-3 was quantified in the right. *P < 0.05, **P < 0.01 and ***P < 0.001

The decrease in FA consumption in the mitochondria could lead to an accumulation of cytosolic lipids and lipotoxicity in IC2-treated cancer cells. Therefore, we speculated that LD formation stimulated by IC2 might function as a protective mechanism for cancer cells. To confirm this, we inhibited LD formation in IC2-treated cancer cells and subsequently evaluated the anti-tumor effects of IC2. The primary lipid component in IC2-stimulated LDs was TGs (Fig. 2A), indicating the involvement of DGAT1, which is responsible for TG synthesis during LD formation. When PC3 cells were treated with the DGAT1 inhibitor T863, IC2-stimulated LD formation was inhibited (Fig. 6A), as a result of the reduction in TG concentration (Fig. 6B). In MTT assays, T863 treatment could enhance the inhibitory effects of IC2 on the proliferation of PC3 cells (Fig. 6C). These results suggest that LDs may play a cell-protective role that compromises the anti-tumor effects of IC2 in cancer cells.

To validate the synergistic effects of IC2 in combination with the inhibitor of LD formation, we further investigated the anti-tumor responses induced by IC2 in cancer cells. Our previous study revealed that IC2 treatment stimulates the apoptosis of breast cancer cells [22]. We analyzed the IC2-induced apoptosis of PC3 cells using flow cytometry with PI and Annexin V-FITC staining. In the presence of the DGAT1 inhibitor, IC2 induced a greater extent of cell apoptosis compared to treatment without the inhibitor (Fig. 6D). To validate the apoptosis, we assessed the cleavage of Poly (ADP-ribose) polymerase (PARP) and caspase-3 using Western blotting. The results showed that the DGAT1 inhibitor increased both PARP and caspase-3 cleavage in IC2-treated cancer cells, thereby confirming the enhanced apoptotic response (Fig. 6E).

**Fig. 7:**
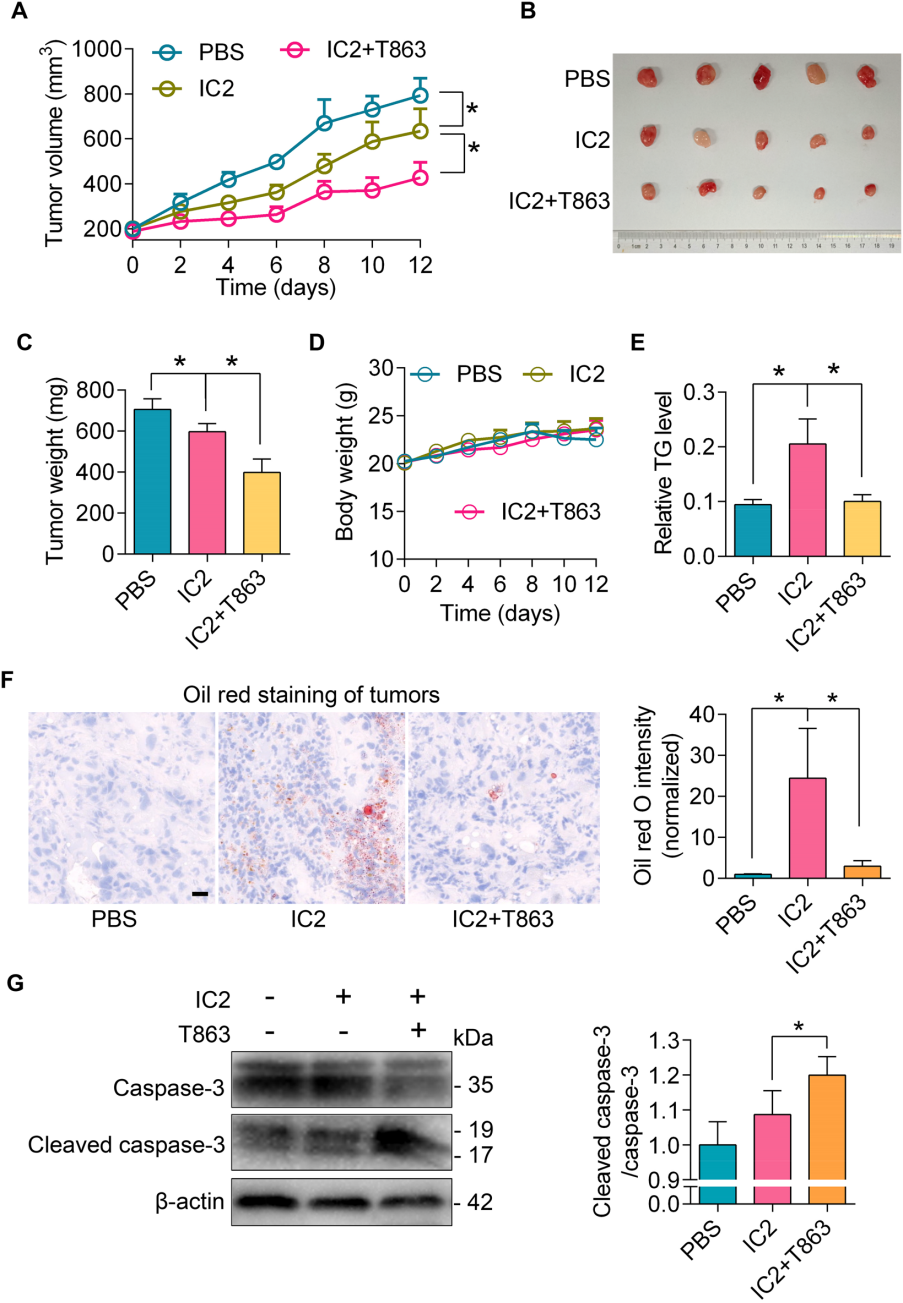
Anti-tumor effects of IC2 combined with an inhibitor of LD formation in vivo. **A** Tumor growth in a xenograft mouse model. The mice were treated with IC2 in the presence or absence of the DGAT1 inhibitor. Tumor volumes were recorded as shown (n = 5). **B** Images of mouse tumors. The tumors were excised from the mice at the end of the experiment. **C** Changes in tumor weights. The tumors were collected as described above (n = 5). **D** Body weights of the mice. Body weight was measured as indicated. No significant differences were observed between the groups. **E** TG contents in tumor samples. TG levels in tumors were measured and normalized to total protein concentrations (n = 5). **F** Oil red O staining of tumor sections. Mice tumors were cryo-sectioned and stained. Representative images of tumors from 5 mice for each group are shown, with staining intensities normalized to the control group. Scale bar = 20 μm. **G** Caspase-3 cleavage in mouse tumors. Caspase-3 and its cleaved form were analyzed by immunoblotting. Representative blots of tumor samples are shown, with blot intensities analyzed on the right (n = 5). *P < 0.05

To validate the findings of our in vitro investigations, we established a PC3 xenograft mouse model and evaluated the combination strategy in an in vivo experiment. IC2 demonstrated a significant inhibition of tumor growth in the xenografted mice, and the addition of the DGAT1 inhibitor T863 further augmented the anti-tumor efficacy of IC2 (Fig. 7A–C). The observed anti-tumor effects were not associated with a reduction in mouse body weight (Fig. 7D). Moreover, IC2 elevated TG contents in mouse tumors, and T863 inhibited this elevation (Fig. 7E). Oil red O staining showed that LD formation in mouse tumors was promoted by IC2, which could be inhibited by T863 (Fig. 7F). Immunoblotting analysis showed that T863 enhanced IC2-stimulated cleavage of caspase-3 in mouse tumors, suggesting the involvement of cell apoptosis (Fig. 7G). These results substantiate that an agent targeting LD formation can synergistically enhance the anti-tumor effects of IC2.

## Discussion

Targeting lipid metabolism in cancer cells, such as the development of SCD1 inhibitors, has been well established for cancer treatment [30, 31]. However, tumor cells may develop adaptive mechanisms to counteract the anti-tumor effects of lipid metabolism-targeting agents [32, 33]. LDs, intracellular organelles for lipid storage, are commonly observed in cancer cells with poor clinical prognoses [34]. LD formation also represents a potential protective mechanism that helps cancer cells alleviate excess oxidative lipotoxicity [34–36]. In this study, we demonstrated that the anti-tumor effects of icaritin derivative IC2 were accompanied by LD generation, and inhibiting LD formation could be developed as a combination strategy for cancer therapy.

The formation of LDs in IC2-treated cancer cells does not occur due to lipid absorption from the extracellular microenvironment. This is evident from the fact that IC2-stimulated LD formation was not halted when the cell medium was deprived of lipids or when a lipid importer inhibitor was used. Moreover, IC2-stimulated LD formation did not depend on de novo FA synthesis in the cancer cells, as inhibition of FASN did not reduce LD formation. In fact, the generation of LDs in IC2-stimulated cells is attributed not to an increase in lipid sources but to a decrease in lipid utilization or consumption. This was supported by lipid distribution analyses using confocal microscopy, which indicated that following IC2 treatment, a larger portion of lipids was located outside of the mitochondria.

IC2-stimulated decrease in lipid utilization was supported by the disrupted functions of mitochondria in cancer cells. This was evidenced by the disrupted MMP and the reduced ATP production capacity, as well as the inhibited oxygen consumption rate observed in IC2-treated cells. Disruptions in mitochondrial functions may result in the cessation of FA beta-oxidation in the cancer cells, which might be the main reason for IC2-stimulated LD formation [37–39]. This conclusion was further supported by the observation that IC2-stimulated LD formation was attenuated by the ACC inhibitor, TOFA. TOFA could decrease the inhibition of ACC2 on FA oxidation in the mitochondria [27, 28]. While this inhibitor also impacts ACC1, which catalyzes FA synthesis, this effect may not be associated with LD formation in the context of IC2 stimulation, as inhibition of FA synthesis by the FASN inhibitor did not affect LD formation.

**Fig. 8:**
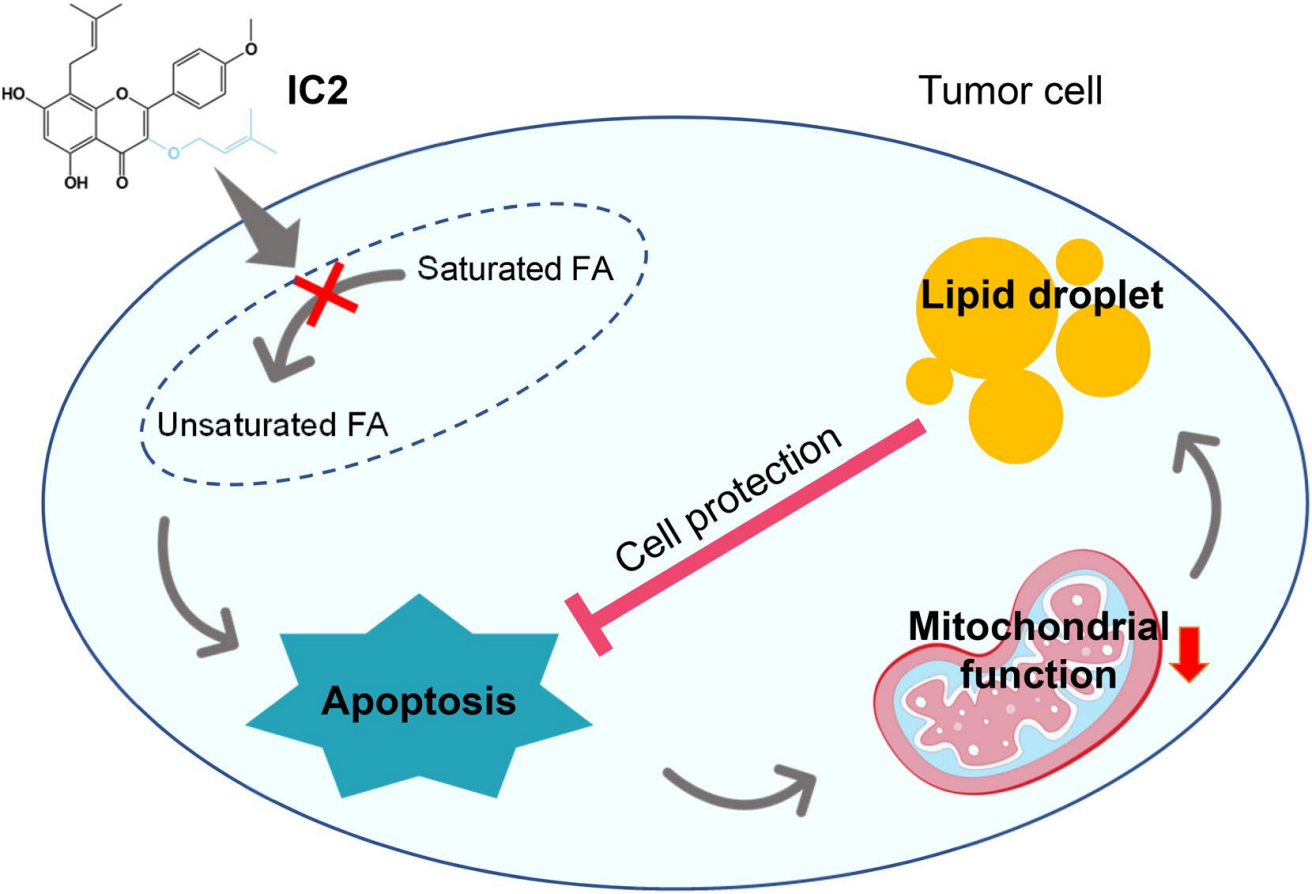
Mechanisms of LD formation with potential cell protective roles in IC2-treated tumor cells. IC2 disrupts the balance between saturated and unsaturated fatty acids (indicated by a cross mark) by targeting SCD1, leading to apoptosis and various responses in tumor cells. The resulting consequences include mitochondrial dysfunctions (indicated by a downward arrow), which promote LD formation that can protect cells from lipotoxicity. LD formation is proposed as a protective mechanism that allows tumor cells to resist the anti-tumor actions of IC2

The present study did not fully elucidate all the mechanisms by which IC2 promotes LD formation in cancer cells. However, when combined with findings from previous studies, we can form a comprehensive understanding of the phenomenon (Fig. 8). IC2 targets SCD1 and inhibits the desaturation step in the process of FA synthesis, which results in the imbalance between saturated and unsaturated FAs in cancer cells [22]. This imbalance poses a stress on the cells, as lipid homeostasis is crucial for cell survival, leading to the activation of intracellular sensors like AMPK [40–42]. These sensors stimulate signaling pathways that trigger various cellular responses, including cell apoptosis and cytoprotective autophagy [23, 43]. The autophagy of mitochondria may also be stimulated and the disruption in mitochondrial function, which is related to lipid consumption, is possibly the initial step of the mitophagy [16, 44, 45]. The reduction of lipid consumption in mitochondria leads to LD formation in the cancer cells, which seems to protect cells from lipotoxicity due to the lipid homeostasis disturbance caused by IC2 stimulation [22, 23].

Our data showed that LD formation was associated with the anti-tumor effects of IC2 in various types of cancer cells. This association may not be perceived that IC2 exerted anti-tumor effects through LD formation, as reducing LD formation by a DGAT1 or ACC inhibitor actually enhanced the anti-tumor effects of IC2. The formation of LDs is likely a concurrent event with IC2’s anti-tumor actions that can protect cancer cells from lipotoxicity. This observation suggests a therapeutic strategy of developing agents that inhibit LD formation in combination with anti-tumor agents. Interestingly, lipid deprivation not only increased the anti-tumor effects of IC2 but also led to an increase in LD formation. While this rise in LD formation can be readily explained as a concurrent event with the enhanced anti-tumor actions of IC2 as above, there remains a possibility that excessive LDs might actively promote the anti-tumor effects of IC2. At least, the possibility that the release of some lipid signals from LDs that promote the apoptosis or ferroptosis, in this context, cannot be excluded [46]. This hypothesis warrants further investigation to confirm its validity. If proven true, an alternative combination therapy that increases excessive LD formation alongside anti-tumor agents could also be developed.

## Conclusions

In conclusion, LD formation in cancer cells is the consequence of the anti-tumor actions of icaritin derivative IC2. The presence of LDs compromises the anti-tumor effects of IC2, highlighting the strategy of combining IC2 with a DGAT1 inhibitor to enhance its effectiveness. The disruption of mitochondrial function is a key mechanism underlying IC2-induced LD formation, indicating the potential of targeting mitochondrial function in combination strategies. It is conceivable that the anti-tumor effects of other lipid metabolism-targeting agents could be enhanced by combining them with agents that target LD formation. This strategy may improve therapeutic outcomes in cancer patients.

## Data Availability

The datasets supporting the conclusions of this article are included within the article and other related information are available from the corresponding author on reasonable request.

## Abbreviations

ACC: Acetyl-CoA carboxylase
AMPK: Adenosine monophosphate-activated protein kinase
BODIPY-LD: BODIPY 493/503
CMXRos: Chloromethyl-X-rosamine
DGAT: Diacylglycerol acyltransferase
FA: Fatty acid
FASN: FA synthase
LD: Lipid droplet
MMP: Mitochondrial membrane potential
MTT: 3-(4, 5-Dimethylthiazol-2-yl)-2,5-diphenyltetrazolium bromide
PARP: Poly (ADP-ribose) polymerase
PGC-1α: Proliferator-activated receptor-γ co-activator 1α
Red-C12: BODIPY558/568-C12
SCD: Stearoyl-CoA desaturase
SREBP1: Sterol regulatory element-binding protein 1
SSO: Sulfosuccinimidyl oleate
T-CHO: Total cholesterol
TG: Triacylglycerol
TOFA: 5-(Tetradecyloxy)-2-furoic acid
TFAM: Mitochondrial transcription factor A
PCa: Prostate cancer
